# DNA Supercoiling Drives a Transition between Collective Modes of Gene Synthesis

**DOI:** 10.1103/PhysRevLett.127.218101

**Published:** 2021-11-19

**Authors:** Purba Chatterjee, Nigel Goldenfeld, Sangjin Kim

**Affiliations:** Department of Physics and Center for the Physics of Living Cells, University of Illinois at Urbana-Champaign, Loomis Laboratory of Physics, 1110 West Green Street, Urbana, Illinois 61801, USA and Carl R. Woese Institute for Genomic Biology, University of Illinois at Urbana-Champaign, 1206 West Gregory Drive, Urbana, Illinois 61801, USA

## Abstract

Transcription of genes can be affected by both biochemical and mechanical factors. Recent experiments suggested that the mechanical stress associated with transcription-induced DNA supercoiling is responsible for the transition from cooperative to antagonistic group dynamics of RNA polymerases (RNAPs) upon promoter repression. To underpin the mechanism behind this drastic transition, we developed a continuum deterministic model for transcription under torsion. In our model, the speed of an RNAP is affected by the local DNA supercoiling, as well as two global factors: (i) the number of RNAPs on the gene affecting the torsional stress experienced by individual RNAPs and (ii) transcription factors blocking the diffusion of DNA supercoils. Our minimal model can successfully reproduce the experimental findings and helps elucidate the interplay of mechanical and biological factors in the collective dynamics of molecular machines involved in gene expression.

Transcription by the molecular machine, RNA polymerase (RNAP) is the first step of gene expression and is known to proceed through a collective mechanism: RNAPs concurrently transcribing a gene increase their efficiency through cooperative interactions [1–4]. Previous experimental and theoretical studies generally predict that the speed of an RNAP (elongation rate) increases with the density of RNAPs, or the rate at which RNAPs initiate at the promoter (RNAP flux) [1–9]. On the contrary, a recent study on the transcription of *lacZ* gene in the bacterium *Escherichia coli* showed that multiple RNAPs translocate at high speed irrespective of the initiation rate, as long as the RNAP flux is continuous (cooperative mode) [10]. Surprisingly, environmentally induced repression, that is an interruption of the RNAP flux, results in a drastic slowdown of co-transcribing RNAPs (antagonistic mode), and this slowdown is more pronounced for later repression (higher RNAP density on the gene).

The transition from cooperative to antagonistic dynamics is likely mediated by DNA supercoiling because it was observed in topologically constrained DNA (e.g., plasmids and chromosomes), but not in linear DNA, where the two ends can freely rotate to dissipate supercoils [10]. How does DNA supercoiling generate these two contrasting modes of RNAP group dynamics? The coupling between DNA supercoiling and RNAP translocation is known for a single RNAP. Forward translation of an RNAP results in underwinding of the DNA behind (negative supercoiling) and overtwisting of the DNA in front (positive supercoiling) [11]. Also, the accumulation of these supercoils slows down the RNAP due to torsional stress [12–14]. However, the effect of DNA supercoiling on multiple RNAPs concurrently transcribing the same DNA is less clear. The simple assumption that positive and negative DNA supercoils cancel commensurately between RNAPs [11,15,16] cannot explain the existence of both cooperative and antagonistic modes of RNAP dynamics.

The density and flux of RNAPs on a gene are important parameters for gene regulation. They vary widely from gene to gene [17–19] and change dynamically with a changing environment through the binding or unbinding of transcription factors (TFs) [20,21]. How DNA supercoiling regulates RNAP dynamics under a wide range of RNAP flux conditions found in physiological settings remains poorly understood.

The purpose of this Letter is to introduce a minimal deterministic model that is generally applicable for describing RNAP translocation dynamics coupled with transcription-induced DNA supercoiling under different RNAP flux conditions. Our model is based on two novel hypotheses regarding the mechanism of torsional-stress generation during transcription. The first hypothesis is that the stress due to DNA supercoiling is exacerbated by the number of RNAPs on the gene. The second hypothesis posits that TFs, which bind near the promoter, affect transcription initiation as well as the diffusion of DNA supercoils. Despite its simplicity, the minimal model accurately recapitulates the experimental observations of Ref. [10] and is a step towards a quantitative understanding of collective effects during gene expression.

## Model.—

**FIG. 1.**
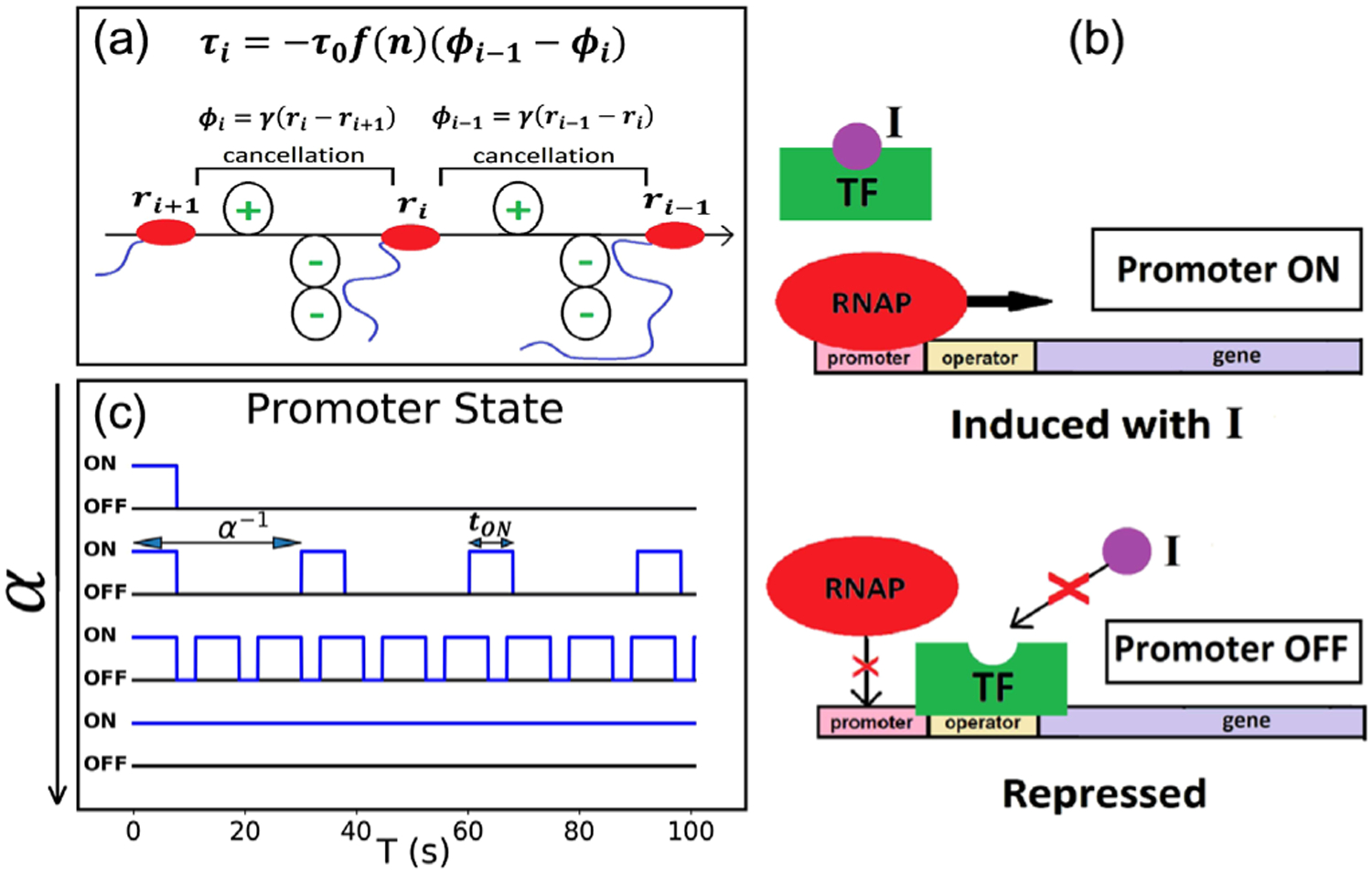
The model. (a) Excess negative supercoiling in DNA segments and restoring torque on RNAP_*i*_. (b) ON and OFF states of the promoter regulated by a repressor TF. (c) Time series of the promoter state for four different initiation rates *α*.

We model the *i*th RNAP (RNAP_*i*_) as a point particle translocating on a gene of length *L* with its position on the DNA given by *r*_*i*_ and speed by $v_{i}={\dot{r}}_{i}$ [Fig. 1(a)]. As it translocates, the RNAP synthesizes an mRNA of increasing length and twists the DNA to generate positive supercoils in front and negative supercoils behind [11]. Positive and negative DNA supercoils are assumed to cancel commensurately between RNAPs [11,15,16]. Hence, the excess negative supercoils introduced into the segment of DNA upstream of RNAP_*i*_, after cancellations with supercoils made by RNAP_*i*+1_, is given by

(1) $$\phi_{i}=\gamma \left(r_{i}-r_{i+1}\right)$$

where *γ* is the rate of supercoil injection per base pair (bp) transcribed by the RNAP. The theoretical maximum for *γ* is 1*/l*_0_ bp^−1^, where *l*_0_ = 10.5 is the number of base pairs in one helical turn of relaxed DNA. However, the actual rate of supercoil accumulation can be much lower (see Discussion), and we choose *γ* = 0.01.

The restoring torque applied by a segment of DNA is taken to be proportional to its excess supercoiling *ϕ* (local effect) [11,22,23]. Also, we hypothesize that this restoring torque depends on the number of RNAPs on the gene, *n* (global effect). This is because having many bulky RNAP molecules on the gene, along with increasing lengths of nascent mRNA synthesized per RNAP, would make it harder to twist the DNA. The net torque acting on RNAP_*i*_ is the difference between the restoring torques applied by its downstream and upstream DNA segments and is given by

(2) $$\tau_{i}=-\tau_{0}f(n)\left(\phi_{i-1}-\phi_{i}\right).$$

Here, *τ*_0_ is a proportionality constant, and *f*(*n*) is a monotonically increasing function of *n*. If the upstream DNA segment is more negatively supercoiled than the downstream DNA segment, the positive net torque provides resistance to translocation [13]. Because of *f*(*n*), the number of RNAPs on the gene can exacerbate the torque effect. This model feature is supported by the observation that repressing the promoter earlier, when there are fewer RNAPs on a gene, yields less slowdown of RNAPs, likely due to smaller resisting torque [10].

The speed of RNAP_*i*_ decreases with increasing torque as

(3) $$v_{i}\left(\tau_{i}\right)=\frac{2v_{0}}{1+\text{exp}\left[2{\left(\tau_{i}/\tau_{c}\right)}^{3}\right]},$$

where *v*_0_ is the typical RNAP speed, and *τ*_*c*_ is the stalling torque, above which RNAP can be halted. The speed depends on *τ*^3^ to ensure that the torsional stress experienced by an RNAP is more pronounced for higher absolute values of the restoring torque, corresponding to higher levels of supercoiling. This also reflects in part the increasing drag on an RNAP as it synthesizes longer mRNA. While the speed drops to zero at high positive torques, negative torques assist transcription elongation [13]. Thus, we posit that an RNAP can transcribe at its maximum speed of 60 bp/s [1,24] under high negative torques.

In our model, RNAP flux is affected by a repressor TF binding at the promoter, which can sterically hinder initiation [Fig. 1(b)]. The promoter can be turned ON when an inducer (*I*) binds to TF and causes it to dissociate. The higher the concentration of *I*, the more frequently TF unbinds, and the higher is the RNAP flux or the transcription initiation rate *α*. If the inducer disappears, TF rebinds on the DNA, and prevents further loading of RNAPs, interrupting RNAP flux (promoter repression).

Following previous experimental observations [25,26], we hypothesize that TF binding, besides turning the promoter OFF periodically [Fig. 1(c)], can also physically block the diffusion of supercoils (see Discussion). When the promoter is turned ON by TF dissociation, the negative supercoils behind the last loaded RNAP would diffuse out, removing torsional stress on this RNAP. We assume that this diffusion takes place before the next RNAP loading event because the diffusion of DNA supercoils is about 100 times faster than RNAP initiation and elongation dynamics [27]. The promoter remains ON for a duration *t*_ON_, which is the average time taken by TF to rebind. As long as the promoter remains ON, no DNA supercoils accumulate behind the last loaded RNAP. When the promoter is turned OFF, the TF blocks both the dissipation of DNA supercoils and the loading of RNAPs till the next time the promoter is turned ON. Repression at time *T*_stop_ turns the promoter OFF completely thereafter and prevents further RNAP loading. Lastly, we allow positive supercoils in front of RNAP_1_ to diffuse downstream unhindered; however, the relaxation of this assumption does not change the main results of the model.

## Model parameters and methods.—

As a proof of concept, we apply our general model to the *lac* operon in *E. coli*, a paradigm of bacterial gene regulation, for which experimental results are available from Ref. [10]. We focus on the transcription of *lacZ*, the first gene in the *lac* operon, with length *L* = 3072 bp. LacI repressor is the TF.

**FIG. 2.**
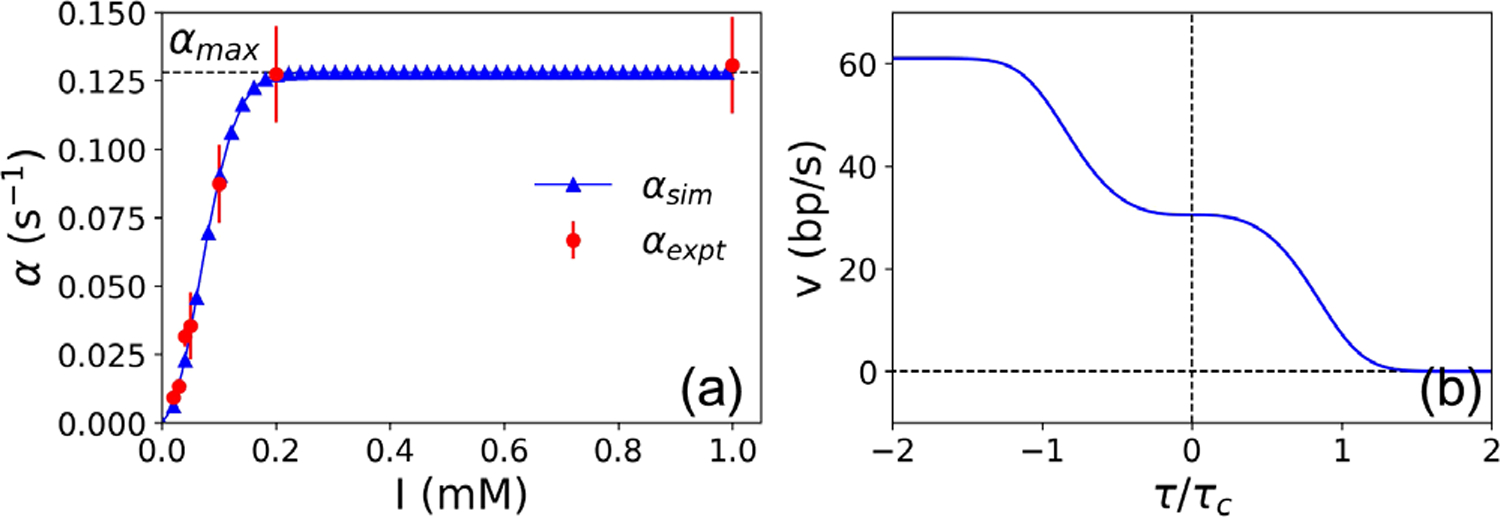
Model parameters. (a) Initiation rates *α* used in the simulations (blue triangles) based on experimental data (red solid circles) in Ref. [10]. (b) Speed of an RNAP *v* as a function of torque *τ/τ*_*c*_.

The effective initiation rates *α*_sim_ used in the simulations were obtained from a fit to the observed *α*_expt_ as a function of the concentration of inducer (*I*) used in Ref. [10] [Fig. 2(a)]. *t*_ON_ is taken to be the inverse of *α*_max_, the highest RNAP flux observed experimentally. We approximate the dependence of the restoring torque on RNAP density by a cubic polynomial of the form *f*(*n*) = 1 + *a*(*n* – 1) + *b*(*n* – 1)^2^ + *c*(*n* – 3)^3^ (see Discussion), with (*a*, *b*, *c*) = (0.78, 3.32, 0.38). Figure 2(b) shows the dependence of RNAP speed *v* on the restoring torque *τ*, where *v*_0_ = 30.5 bp/s is the typical RNAP speed [10], *τ*_*c*_ = 11 pN · nm is the stalling torque measured for *E. coli* RNAP [13], and *τ*_0_ = 0.386 pN · nm. A different value of stall torque [28] would only require a different choice of *τ*_0_, without changing our main results.

To calculate the average elongation rate of the first RNAP (RNAP_1_), we follow the prescription in Ref. [10]. When the promoter is active, the average elongation rate is *v*_ON_ = *L/T*_1_, where *T*_1_ is the time taken by RNAP_1_ to complete transcription. When the promoter is repressed at time *T*_stop_, the average elongation rate of RNAP_1_ for the remaining portion of the gene after repression is *v*_OFF_ = (*L* − *v*_ON_*T*_stop_)*/*(*T*_1_ − *T*_stop_). This definition of *v*_OFF_ assumes that the RNAPs move at a constant speed *v*_ON_ till the promoter is repressed at *T* = *T*_stop_. The assumption is not always valid (see Supplemental Material, Fig. S1 [29]), but we adhere to this definition of *v*_OFF_ for comparison with the experimental results of Ref. [10].

## Simulation results.—

**FIG. 3.**
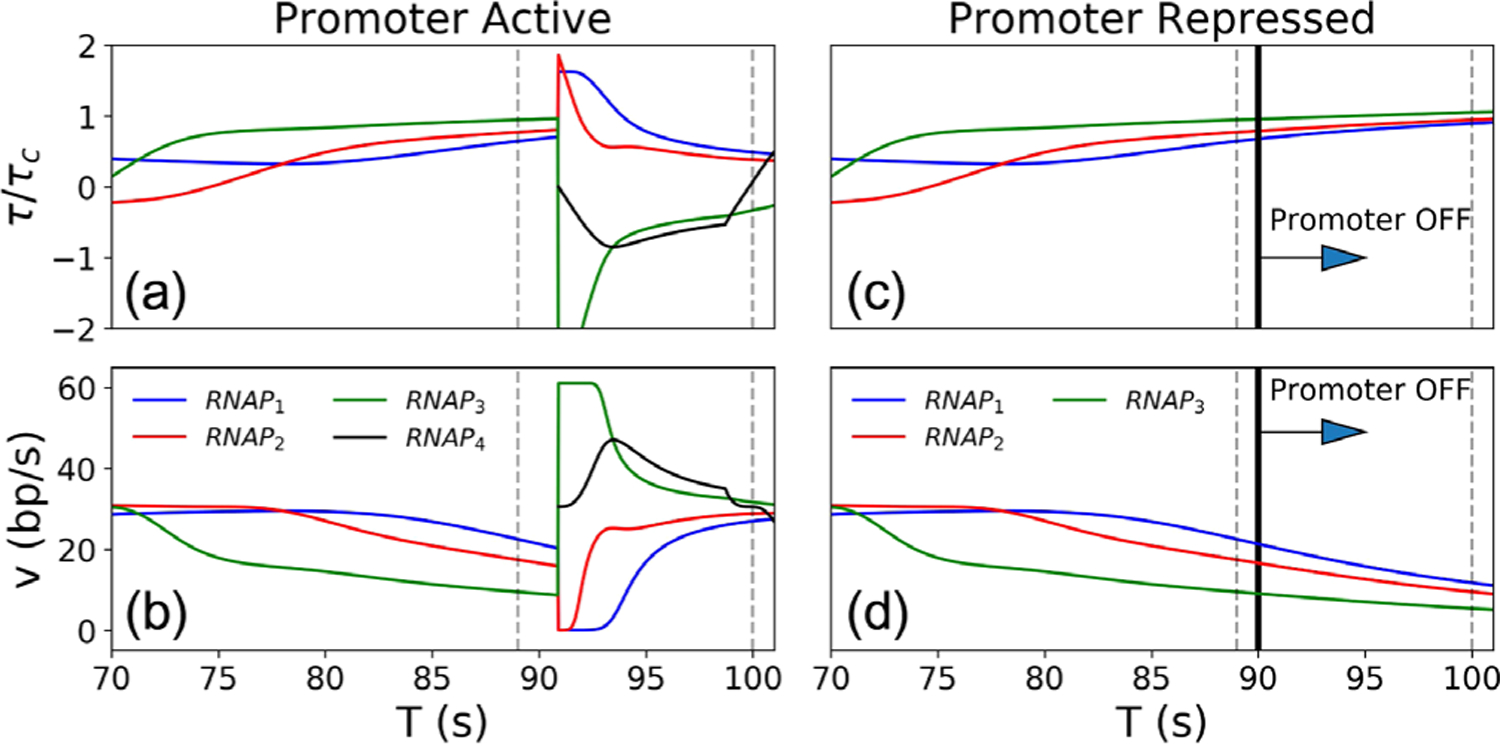
Time series of *τ/τ*_*c*_ and *v* of the first 3–4 RNAPs for the case of active promoter (a)–(b) and promoter repression at *T* = 90 s (c)–(d) at the intermediate initiation rate *α* = 0.033 s^−1^. We only plot *τ/τ*_*c*_ ∈ (−2, 2) for clarity. Gray dashed lines demarcate the time range for Supplemental Material, Fig. S2 [29].

Figures 3(a) and 3(b) show the time series of *τ*/*τ*_*c*_ and *v* for the first 3–4 RNAPs at the intermediate initiation rate *α* = 0.033 s^−1^, in which the promoter cycles between ON and OFF states [Fig. 1(c) second from top]. At *T* = 70 s, there are three RNAPs on the gene moving at *v*_0_ bp/s. However, because the promoter is in the OFF state, DNA supercoil diffusion is blocked at the promoter by TF, leading to a sequential slowing down of RNAPs starting with RNAP_3_, until the next TF dissociation event (at *T* ≈ 91 s). At this time, the negative supercoils behind RNAP_3_ dissipate, and all RNAPs on the gene can quickly equilibrate to the optimal speed *v*_0_ by *T* = 100 s. This equilibration proceeds through the acceleration and deceleration of RNAPs reacting to the ambient torsional stress (detailed in the Supplemental Material, Fig. S2 [29]).

If the promoter is repressed at *T* = 90 s [Figs. 3(c) and 3(d)], TF remains bound (RNAP_4_ does not load), and the speeds of RNAP_1_, RNAP_2_, and RNAP_3_ continue to decrease. The speed of RNAP_1_ is reduced to 11.68 bp/s at *T* = 100 s, and it decreases even further thereafter. Therefore, promoter repression in the model recapitulates RNAP slowdown observed in the experiments (For the dynamics at low and high initiation rates, see Supplemental Material, Figs. S1 and S3 [29]).

**FIG. 4.**
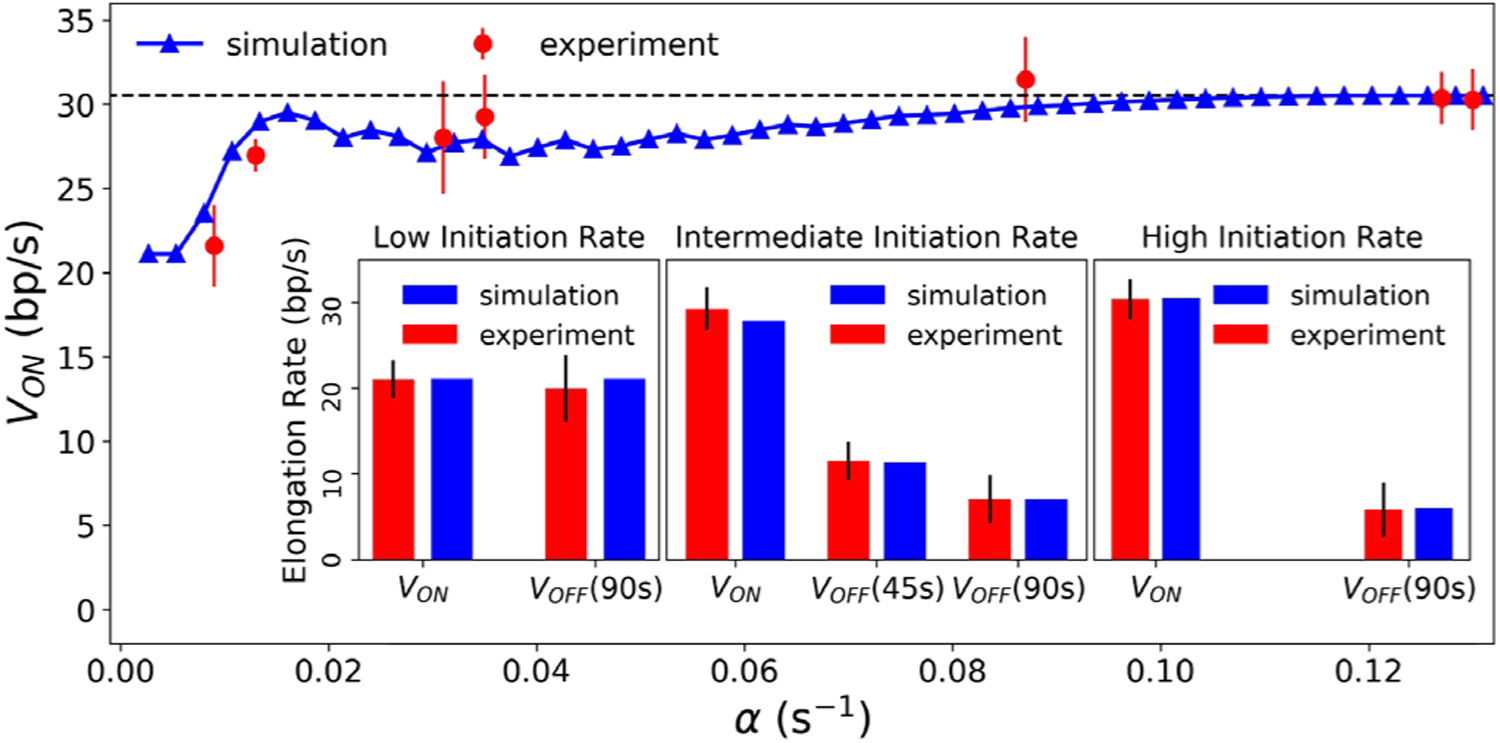
Comparison of our model (blue) with the experimental results of Ref. [10] (red) on the speed of RNAPs *v*_ON_ as a function of initiation rates *α*. The inset shows the effect of promoter repression. Low initiation rate represents *α*_sim_ = 0.006 s^−1^ (*α*_expt_ = 0.009 s^−1^), yielding roughly a single RNAP on the gene at a given time. Intermediate and high initiation rates are from *α*_sim_ = 0.033 s^−1^ (*α*_expt_ = 0.035 s^−1^) and *α*_sim_ = 0.127 s^−1^ (*α*_expt_ = 0.127 s^−1^), respectively.

Figure 4 shows the average elongation rate *v*_ON_ vs initiation rate *α*. For low initiation rates (*α*_sim_ = 0.006 s^−1^), there is only a single RNAP on the gene on average, and *v*_ON_ is less than the typical speed *v*_0_ = 30.5 bp/s. However, for a large range of higher *α* values, *v*_ON_ ≈ *v*_0_, independent of the initiation rate. The inset of Fig. 4 shows the average elongation rate *v*_OFF_ upon promoter repression for three different initiation rates tested in the experiments. At a low initiation rate (*α*_sim_ = 0.006 s^−1^), only a single RNAP transcribe a gene at a time, and promoter repression at *T* = 90 s does not appreciably affect the RNAP speed. For intermediate (*α*_sim_ = 0.033 s^−1^) and high (*α*_sim_ = 0.127 s^−1^) initiation rates, promoter repression at *T* = 90 s causes speeds to drop to about a quarter of a single RNAP speed. Notably, the effect is smaller if the promoter is repressed earlier (e.g., at *T* = 45 vs *T* = 90 s for the intermediate initiation rate), consistent with the experimental observation [10].

## Discussion.—

Our model generates three distinct modes of transcription based on RNAP flux and mechanical stress. For a continuous flux of RNAPs (active promoter), a collective cooperative mode is observed, where co-transcribing RNAPs can efficiently cancel each other’s supercoils and move at optimal speeds, making many transcripts at a given time. When this flux is interrupted (promoter repression), we observe a collective antagonistic mode, characterized by a drastic reduction in the speed of co-transcribing RNAPs due to supercoil accumulation. The reduction is more pronounced for later repression, or if the repression occurs when the RNAPs are closer to the transcription termination. The slowdown may yield a sudden brake in transcription, especially when the slowdown results in pre-mature dissociation of RNAPs [10]. Lastly, for very low fluxes (a single RNAP), we observe a torsionally stressed mode of transcription elongation with lower than optimal speeds, but it is not altered by promoter repression. This likely corresponds to a basal level of transcription that is not actively regulated.

The total inertial resistance to DNA twisting is a product of the total mass of RNAPs on the gene as well as that of the increasing lengths of nascent mRNAs. While the former increases linearly with *n*, the total length of mRNA synthesized increases as *R* ∝ *n*(*n* – 1)*/*2 ≈ *n*^2^. Therefore, we approximate the dependence of the restoring torque on RNAP density, *f*(*n*), with a cubic polynomial. However, the exact functional form for *f*(*n*) and the coefficients are likely tied to the exact experimental conditions under consideration since they determine the twist modulus of DNA. Additionally, we choose a constant supercoil injection rate *γ* = 0.01, of the order of the approximate supercoiling density (number of supercoils per unit length of DNA transcribed) reported in the literature [11,22,23,30] because the actual supercoil injection rate *in vivo* remains unknown. It can be affected by various factors (e.g., frictional drag, topoisomerase activity, downstream topological barriers, supercoil diffusion, and DNA stretching forces), and it may change with the position of RNAP on the gene [11,22,23,30].

Our hypothesis that TFs can regulate the diffusion of DNA supercoils is supported by the observations that LacI functions as a topological barrier to constrain DNA supercoils [25,26]. This hypothesis allows for coupling the RNAP flux with the dissipation of DNA supercoils. We note that other molecules, such as RNAPs poised at the promoter, may have a similar effect as TFs [10,31,32]. Another novel implication of this hypothesis is for modeling transcription dynamics in the genomic context, where DNA supercoiling produced from neighboring genes should be considered [33,34]. For example, the diffusion of DNA supercoils or its lack thereof likely has important consequences for divergently transcribed genes commonly found in the genome [35]. It was shown in Ref. [10] that the divergent expression of another gene, positioned upstream of *lacZ*, reduces the transcription elongation rate of *lacZ* in the case of the high initiation rate *α* = 0.127 s^−1^. Moreover, this antagonistic effect was observed even when the two promoters are separated by as much as 2400 bp. This is entirely consistent with our model’s prediction. At high initiation rates, the promoter is almost always ON [Fig. 1(c) highest *α*]. As a result, negative DNA supercoils generated by transcription of a neighboring gene can diffuse in and reduce the speed of RNAPs transcribing *lacZ*.

**FIG. 5.**
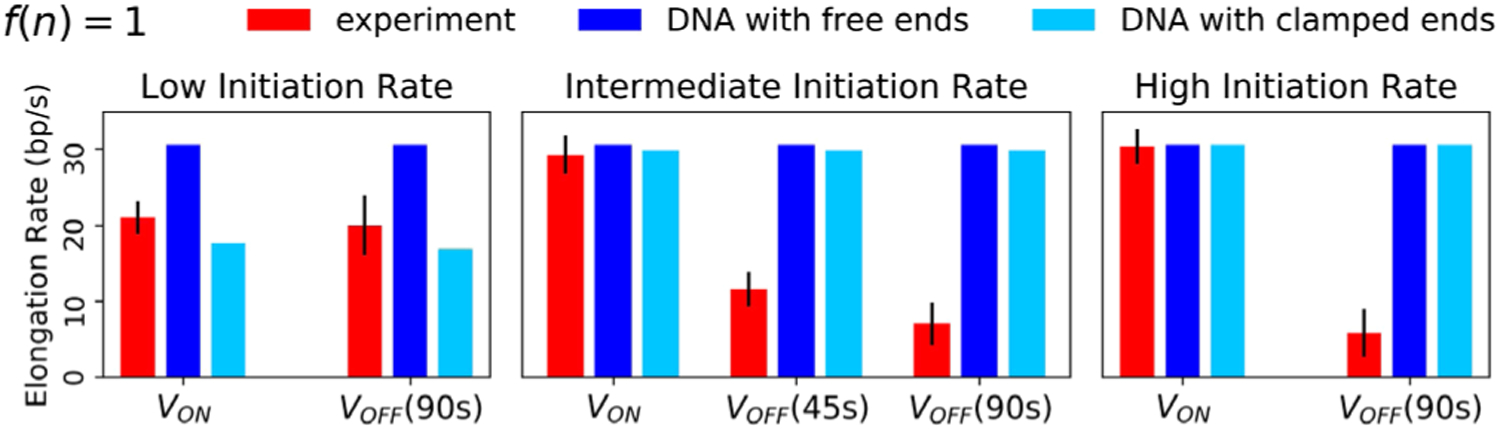
Consequences of relaxing the central assumptions of our model. Red and blue bars represent experimental data and simulation results from *f*(*n*) = 1, respectively. (Dark blue) Free DNA: TF does not constrain supercoils even when bound near the promoter. (Light blue) Clamped DNA: supercoils are always constrained near the promoter, even when TF is unbound.

Our model is different from existing theories of transcription based on DNA supercoiling [5,8,23,36,37] because it considers not only transcription-induced DNA supercoils (local effect) but also the role of TFs as a barrier for the DNA supercoil diffusion and the dependence of the DNA restoring torque on the number of RNAPs on the gene (global effects). We find that these model features are critical in producing the two contrasting RNAP group dynamics between the active and repressed states of the promoter. Without them, it is not possible to consistently explain the experimental findings *even qualitatively* within our framework (details in Supplemental Material, Figs. S4–S7 [29]). For example, Fig. 5 demonstrates the consequences of relaxing both of the central assumptions of our theory. Here, we consider two scenarios in which the restoring torque does not depend on RNAP density [*f*(*n*) = 1]. In one case, TF does not constrain DNA supercoils when bound (similar to linear DNA whose ends are free to rotate and dissipate torsional stress). In this case, high speeds are observed for all initiation rates, even for a single RNAP, and moreover, the switch to antagonistic dynamics upon promoter repression cannot be recovered. In the other case, DNA supercoils are always constrained independent of TF binding (similar to DNA with clamped ends). Here, the collective cooperative mode is recovered, with multiple co-transcribing RNAPs translocating faster than a single RNAP when the promoter is active. However, their mutual antagonism upon promoter repression cannot be captured. We have also investigated the effect of imposed bursty initiation and found that contrary to existing literature [38], our model predicts that DNA supercoiling hinders the formation of convoys of RNAPs traveling at the same speed (see Supplemental Material, Fig. S8 [29]), even with bursty loading.

## Supplementary Material

SuppText
