# Supplementary material for: DNA Supercoiling Drives a Transition between Collective Modes of Gene Synthesis
Source: Phys Rev Lett. Author manuscript; Available in PMC 2022 Apr 23. (PMC9034659; doi:10.1103/PhysRevLett.127.218101)
Supplement: SuppText [file NIHMS1798982-supplement-SuppText.pdf]

Supplementary Material for: DNA Supercoiling Drives a Transition between Collective Modes of Gene Synthesis

Purba Chatterjee, Nigel Goldenfeld, and Sangjin Kim
Department of Physics and Center for the Physics of Living Cells,
University of Illinois at Urbana-Champaign, Loomis Laboratory of Physics,
1110 West Green Street, Urbana, Illinois 61801, USA
Carl R. Woese Institute for Genomic Biology, University of Illinois at Urbana-Champaign,
1206 West Gregory Drive, Urbana, Illinois 61801, USA

In this supplementary material, we provide the details of RNAP dynamics for different initiation rates and promoter activities, i.e., active and repressed promoter (Sec. S1). Furthermore, we try to relax the central hypotheses of our model and compare the results with experimental data (Sec. S2 and Sec. S3). We also investigate the effect of imposed bursty initiation in our model (Sec. S4). Throughout the supplementary materials, we will refer to negative supercoils as NS.

S1. COMPARISON OF DYNAMICS FOR ACTIVE AND REPRESSED PROMOTERS

Here, we compare the torsional stress and speeds of the first few RNAPs for different promoter strength (low, intermediate, and high initiation rates) and activities (active and repressed).

A. Low Initiation Rate

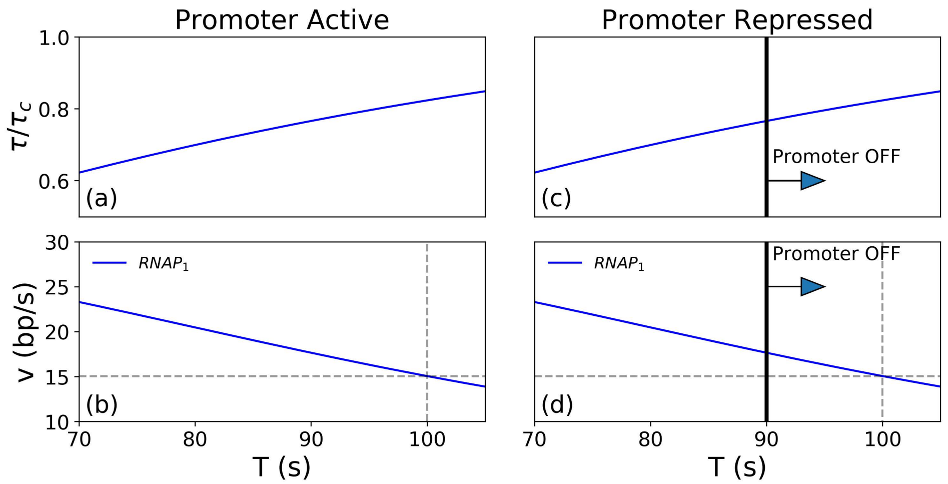

FIG. S1. (color online) Time series of τ/τ_c and v of an RNAP at the low initiation rate $\alpha = 0.006$ s^{-1} when the promoter is active (a-b) and when the promoter is repressed at $T = 90$ s (c-d). The speed of the single RNAP continues to decrease even when the promoter is active (b), and the same RNAP dynamics is seen even when the promoter is repressed $T = 90$ s (d).

At the low initiation rate ($\alpha = 0.006$ s^{-1}), there is only a single RNAP on the gene on average. Fig. S1 shows the time series of restoring torque τ/τ_c and speed v of the single RNAP for the active promoter (Fig. S1(a,b)) and for the promoter repressed at $T = 90$ s (Fig. S1(c,d)). The speed decreases continuously, such that at $T = 100$ s we have $v = 15.05$ bp/s for both active *and* repressed promoters. The time taken by the single RNAP to complete transcription, T_1, is the same in both cases. Due to low α, there is no upstream RNAP that can assist through DNA supercoil cancellation. Thus, the dynamics of a single RNAP is subject to similar levels of torsional stress irrespective of the promoter state.

Because the RNAP speed decreases continuously at low initiation rates, the definition of the average elongation rate after repression in [13], $v_{OFF} = (L - v_{ON}T_{stop})/(T_1 - T_{stop})$, underestimates the position of the single RNAP

upon repression (i.e., when $T = T_{stop}$). As a result, it predicts a higher v_{OFF} than if we were to consider the actual position $r_1(T_{stop})$. This is a limitation of the experiment, which does not track the position of the RNAPs. One has to assume a constant speed v_{ON} till T_{stop} in order to calculate the speed after repression, v_{OFF}.

Whenever the time taken to complete transcription with the active promoter (T_{end}^{ON}) is equal to that with the promoter repressed at T_{stop} (T_{end}^{OFF}), this definition of v_{OFF} always predicts $v_{OFF} = v_{ON}$. That is, with $L = v_{ON}T_{end}^{ON}$, we have

$$\begin{aligned} v_{OFF} &= \frac{L - v_{ON}T_{stop}}{T_{end}^{OFF} - T_{stop}}, \\ &= \frac{v_{ON}(T_{end}^{ON} - T_{stop})}{T_{end}^{OFF} - T_{stop}}, \\ \implies v_{OFF} &= v_{ON}, \end{aligned} \tag{S1}$$

for $T_{end}^{OFF} = T_{end}^{ON}$. The actual elongation rate after repression would be lower than that calculated by this prescription, on average. However, we chose to adhere to this definition of v_{OFF} for accurate comparison with the experimental results of [13]. What is important to note is that the dynamics of the RNAP remains unaffected by the promoter state and that the time taken to complete transcription is the same for both active and repressed promoters (regardless of T_{stop}) when there is a single RNAP on the DNA. Thus, one should read the result $v_{ON} = v_{OFF}$ for the single RNAP case as $T_{end}^{OFF} = T_{end}^{ON}$, that is the time of transcription completion for a single RNAP is unaffected by active or repressed conditions of the promoter.

B. Intermediate Initiation Rate

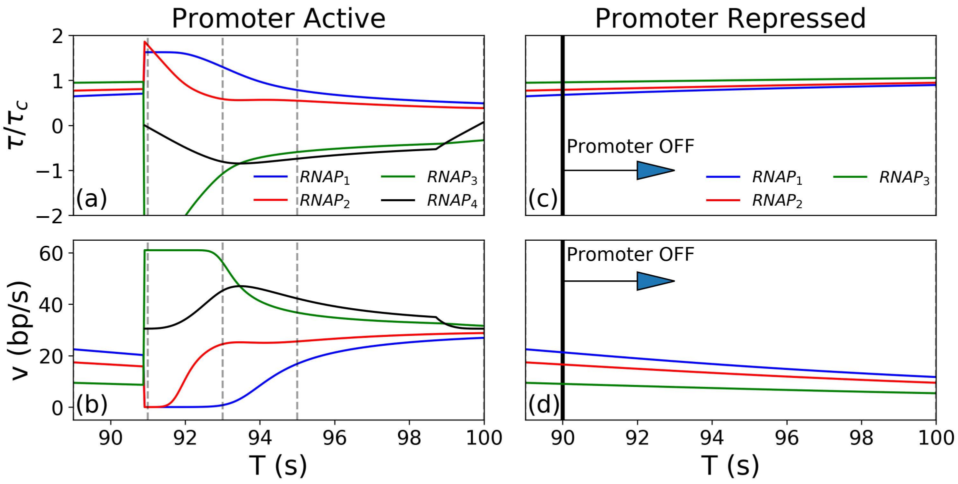

FIG. S2. (color online) Time series of τ/τ_c and v of the first 3 - 4 RNAPs from a promoter with the intermediate initiation rate $\alpha = 0.033$ s^{-1}. (a-b) is when the promoter is continuously active, and (c-d) is when the promoter is repressed at $T = 90$ s. This is a zoom-in version of Fig. 3 of the main text. The dashed gray lines mark the time points discussed below. When the promoter is active, the first three RNAPs start at the typical speed v_0, but their speeds reduce due to NS accumulation. However, $RNAP_4$ loads at $T \approx 91$ s, and the resulting dynamics allows the speed of all four RNAPs to equilibrate to the typical speed v_0 by $T = 100$ s. In contrast, with promoter repression at $T = 90$ s, speeds of the three loaded RNAPs continue decreasing beyond $T = 91$ s.

Fig. S2 shows the dynamics of RNAPs for the intermediate initiation rate $\alpha = 0.033$ s^{-1}. We plotted the time series of restoring torque τ/τ_c and speed v for the first 3 - 4 RNAPs within the time range demarcated by gray dashed lines in Fig. 3 of the main text. At $T = 89$ s, there are three RNAPs on the gene moving at speeds less than v_0, with $RNAP_3$ the slowest and $RNAP_1$ the fastest. $RNAP_3$ is slow because NS accumulates behind it while TF remains bound. There is a sequential slowing down of all downstream RNAPs starting from the promoter region due to insufficient cancellation of their NS by their slow upstream neighbor RNAPs.

When the promoter stays active, TF dissociates for the next RNAP loading. In Fig. S2(a,b), TF dissociates at $T \approx 91$ s for the loading of $RNAP_4$, and there are a few consequences. In our model, when TF dissociates, the NS

behind $RNAP_3$ diffuses out first prior to $RNAP_4$ loading, and hence, the remaining NS *in front* of the $RNAP_3$ causes its speed to increase (green). As $RNAP_4$ loads, the speeds of $RNAP_1$ (blue) and $RNAP_2$ (red) fall, owing to the increased difficulty of translocating by overtwisting the DNA with an additional RNAP on the gene. The fast-moving $RNAP_3$ can cancel supercoils ahead more efficiently, so it speeds up $RNAP_2$ (see $T \approx 92$ s). $RNAP_4$ (black) initially speeds up right after loading because it has NS ahead, owing to the high speed of $RNAP_3$. Thus, at $T = 93$ s, we see both $RNAP_2$ and $RNAP_4$ accelerating. Soon after, at $T = 95$ s, $RNAP_3$ and $RNAP_4$ start slowing down due to NS accumulation behind them, and at the same time, $RNAP_1$ and $RNAP_2$ are speeding up due to better cancellation of their NS. Eventually, at around $T = 100$ s, all four RNAPs have once again settled to the typical speed v_0.

TF rebinds at some point after $RNAP_4$ loading and blocks supercoil diffusion. As more NS accumulate behind $RNAP_4$, it starts to slow down, re-initiating a sequential decrease in the speeds of downstream RNAPs. However, as long as loading is uninterrupted (active promoter), RNAPs can always equilibrate to the optimal speed. In contrast, when the promoter is repressed at $T = 90$ s (Fig. S2(c,d)), $RNAP_4$ does not load, and the speeds of $RNAP_1$, $RNAP_2$, and $RNAP_3$ continue to decrease. At $T = 100$ s, the speed of $RNAP_1$ reduces to 11.68 bp/s, as compared to 26.95 bp/s when the promoter remains active. The RNAP slows down even further after $T = 100$ s to finally record an average elongation rate $v_{OFF} = 7.02$ bp/s, as shown in Fig. 4 of the main text.

C. High Initiation Rate

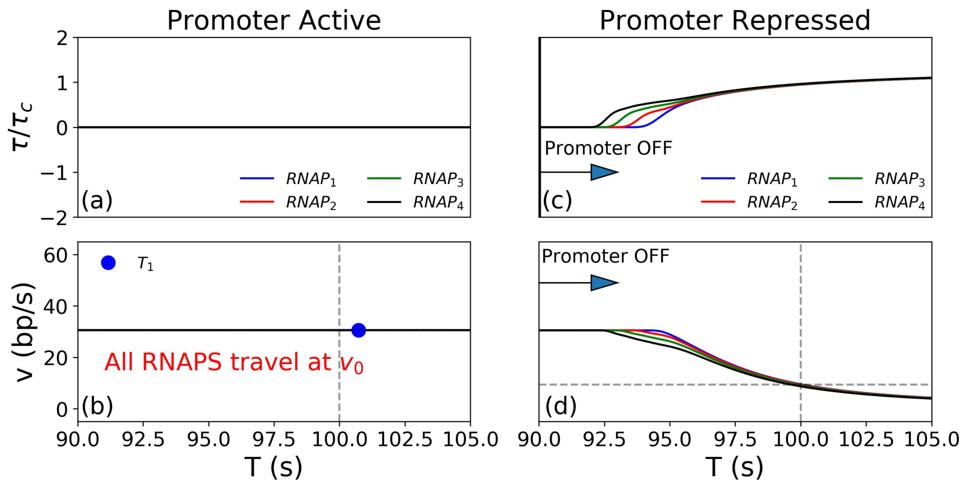

FIG. S3. (color online) Time series of τ/τ_c and v of the first four RNAPs in the case of uninterrupted loading (a-b) and promoter repression at $T = 90$ s (c-d) for the high initiation rate $\alpha = 0.127$ s^{-1}. When the promoter remains active, all RNAPs travel at the typical speed v_0. In contrast, after promoter repression at $T = 90$ s, speeds of the first four RNAPs suddenly reduce drastically over a short period of time as the torsional stress crosses threshold values.

At a high initiation rate ($\alpha = 0.127$ s^{-1}) of the active promoter, TF dissociates frequently, and the promoter is almost always ON (see Fig. 1 for the highest α). As such, NS do not accumulate behind the last loaded RNAP as long as the promoter is active. Fig. S3 shows the time series of τ/τ_c and v of the first four RNAPs for the active promoter (Fig. S3(a,b)) and for the promoter repressed at $T = 90$ s (Fig. S3(c,d)). It is clear that for the active promoter, there is negligible torsional stress throughout, and all RNAPs travel at the typical speed v_0. In Fig. S3(b), we have marked $T_1 = 100.73$ s, the time taken for the first RNAP to complete transcription. In contrast, when the promoter is repressed at $T = 90$ s, we see a drastic reduction of RNAP speeds over a very short period of time. For example, the speed of the first RNAP reduces to $v = 9.48$ bp/s at $T = 100$ s. This reduction is caused by TF binding at T_{stop}, which prevents both further initiation as well as the diffusion of NS produced by the last loaded RNAP. Moreover, because there are approximately $n = 12$ RNAPs on the gene when the promoter is repressed, the accumulation of a very small amount of supercoiling is sufficient to increase the torsional stress beyond threshold values. Thus, promoter repression in the model recapitulates the drastic reduction in RNAP speeds observed in the experiments [13]. A greater reduction is expected for larger RNAP densities on the gene, i.e for higher initiation rates, suggesting that this antagonistic effect is another group effect of RNAPs.

S2. TORSIONAL STRESS INDEPENDENT OF RNAP DENSITY

In our model, we hypothesize that the presence of many RNAPs on the gene exacerbates the torsional stress by making the DNA more difficult to overtwist. The dependence of the restoring torque τ on the RNAP density n is encoded by the function $f(n)$. To relax this assumption in our model, we consider the situation where τ is independent of n, i.e. $f(n) = 1$. In the following subsections, we examine $f(n) = 1$ under three different scenarios related to supercoil diffusion at the promoter.

A. TF Blocks NS Diffusion in its Bound State

Fig. S4 shows elongation rates of various conditions assuming that DNA-bound TF blocks NS diffusion (as in our main model) but $f(n) = 1$. In the active state of the promoter, we once again see high elongation rates independent of initiation rates for intermediate to high α, whereas the single RNAP case at low initiation rates (e.g., $\alpha = 0.006$ s^{-1}) has a lower speed. However, for all initiation rates, promoter repression at neither $T = 45$ s nor $T = 90$ s shows any change in elongation rates v_{OFF} from their v_{ON} values (Fig. S4 (inset)). This is in contrast to the experimental observation of [13] that promoter repression causes a large reduction in RNAP speeds. Thus, without the dependence of torsional stress on RNAP density, even with TF blocking NS diffusion when bound, we cannot reproduce the observed switch from cooperative to antagonistic collective dynamics of RNAPs upon promoter repression.

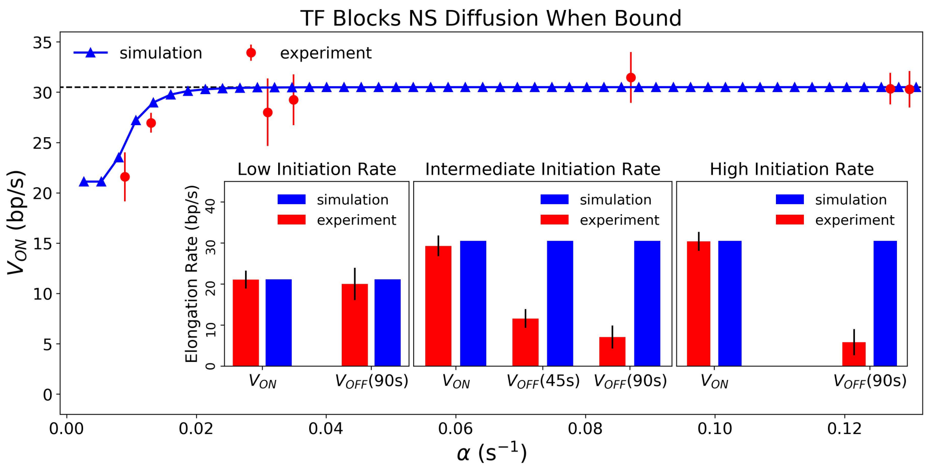

FIG. S4. (color online) Scenario with $f(n) = 1$ and TF blocking NS diffusion in its bound state. v_{ON} is low for a single RNAP ($\alpha_{sim} = 0.006$ s^{-1}), but it remains high independent of initiation rates for a large range of α. The inset shows the effect of promoter repression. Promoter repression at $T = 45$ s or $T = 90$ s does not appreciable change v_{OFF} from v_{ON} for low ($\alpha_{sim} = 0.006$ s^{-1}, $\alpha_{expt} = 0.009$ s^{-1}), intermediate ($\alpha_{sim} = 0.033$ s^{-1}, $\alpha_{expt} = 0.035$ s^{-1}), and high ($\alpha_{sim} = 0.127$ s^{-1}, $\alpha_{expt} = 0.127$ s^{-1}) initiation rates.

B. DNA with Free Ends: TF Never Blocks NS Diffusion

Fig. S5 shows elongation rates for the case where $f(n) = 1$ and TF never blocks NS diffusion. In other words, NS always diffuse out. This situation could arise in the case of linear DNA that always allows supercoil dissipation through its free ends or in the case where TF is a comparatively smaller molecule and cannot constrain supercoils. In the active promoter, we see high elongation rates independent of initiation rates for *all* α. Even a single RNAP ($\alpha = 0.006$ s^{-1}) transcribes at the optimal speed. This is contradictory to the experimental observation that co-transcribing RNAPs can collectively increase their elongation rates in comparison to a single RNAP. Additionally, like in Sec. S2 A, promoter repression at $T = 45$ s or at $T = 90$ s does not show any change in elongation rates for any initiation rate (inset in Fig. S5). Thus, with the torsional stress independent of RNAP density ($f(n) = 1$) and with TF unable to block NS diffusion even when bound, we cannot reproduce either the collective or the antagonistic dynamics of RNAPs observed in [13].

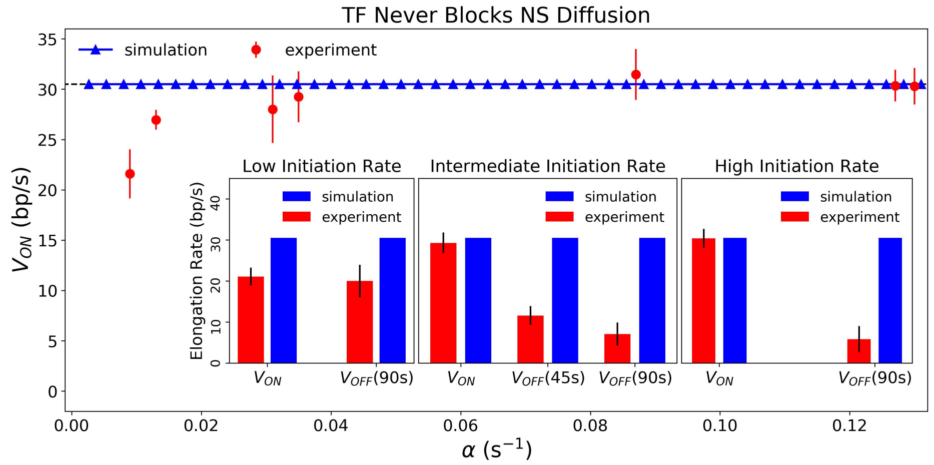

FIG. S5. (color online) Scenario with $f(n) = 1$ and free DNA with TF never blocking NS diffusion. v_{ON} is high and independent of initiation rates for all α, even for a single RNAP ($\alpha_{sim} = 0.006$ s^{-1}). The inset shows the effect of promoter repression. Promoter repression at $T = 45$ s or $T = 90$ s does not appreciable change v_{OFF} from v_{ON} for low ($\alpha_{sim} = 0.006$ s^{-1}, $\alpha_{expt} = 0.009$ s^{-1}), intermediate ($\alpha_{sim} = 0.033$ s^{-1}, $\alpha_{expt} = 0.035$ s^{-1}), and high ($\alpha_{sim} = 0.127$ s^{-1}, $\alpha_{expt} = 0.127$ s^{-1}) initiation rates.

C. DNA with Clamped Ends: No NS Diffusion

Fig. S6 shows elongation rates for $f(n) = 1$ and for DNA clamped at its ends, with no NS diffusion. This case explores the scenario where TF binding or unbinding only affects RNAP loading but is irrelevant to the torsional stress. This can also be considered as the general case with a bulky molecule always bound to the DNA upstream of the promoter, which does not affect RNAP loading but blocks NS diffusion. Fig. S6 shows that elongation rates

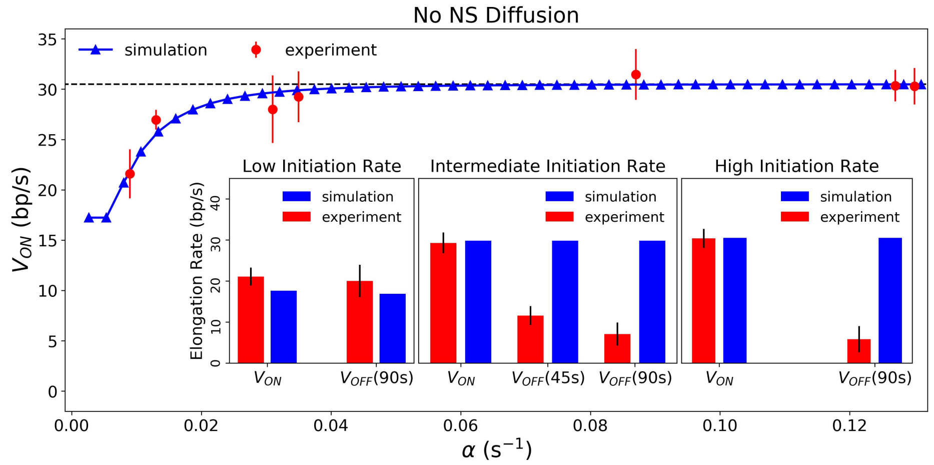

FIG. S6. (color online) Scenario with $f(n) = 1$ and clamped DNA with no NS diffusion. The main panel shows that v_{ON} is low for low initiation rates and high and independent of the initiation rates for intermediate and high α. The inset shows the effect of promoter repression. Promoter repression at $T = 45$ s or $T = 90$ s does not appreciable change v_{OFF} from v_{ON} for intermediate ($\alpha_{sim} = 0.033$ s^{-1}, $\alpha_{expt} = 0.035$ s^{-1}) and high ($\alpha_{sim} = 0.127$ s^{-1}, $\alpha_{expt} = 0.127$ s^{-1}) initiation rates.

are low for low initiation rates but high and independent of α for intermediate to high initiation rates of active promoters. This agrees with the observations of [13]. However, promoter repression at $T = 45$ s or at $T = 90$ s does not show any change in the elongation rates for intermediate and high initiation rate (Fig. S6 (inset)), contrary to [13]. Thus, without n dependence and NS diffusion, we cannot capture the negative effect of promoter repression on the co-transcribing RNAPs.

S3. SUPERCOIL DIFFUSION NOT BLOCKED BY TF

In our main model, we hypothesize that the presence and absence of TF on the DNA imposes different conditions of torsional stress on the transcription elongation dynamics. When TF is bound, it blocks NS diffusion and constrains them between itself and the last loaded RNAP. Unbinding of TF immediately results in the dissipation of this torsional stress, and we say that the NS behind the last loaded RNAP can diffuse out and not affect its speed. Keeping the n dependence of the torsional stress identical to the main text, we tried to relax this assumption in two ways - first by looking at the scenario where NS always diffuse out (free DNA as in Sec. S2 B) and second by considering the case where NS never diffuse out (clamped DNA as in Sec. S2 C).

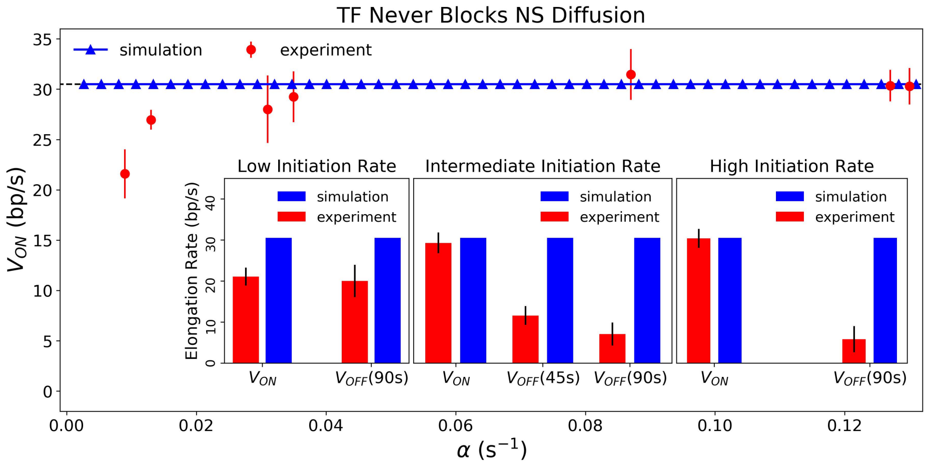

FIG. S7. (color online) Scenario with $f(n)$ as in original model, and free DNA, i.e. TF never blocking NS diffusion. v_{ON} is high and independent of initiation rates for all α, even for a single RNAP ($\alpha_{sim} = 0.006$ s^{-1}). The inset shows the effect of promoter repression. Promoter repression at $T = 45$ s or $T = 90$ s does not appreciable change v_{OFF} from v_{ON} for low ($\alpha_{sim} = 0.006$ s^{-1}, $\alpha_{expt} = 0.009$ s^{-1}), intermediate ($\alpha_{sim} = 0.033$ s^{-1}, $\alpha_{expt} = 0.035$ s^{-1}), and high ($\alpha_{sim} = 0.127$ s^{-1}, $\alpha_{expt} = 0.127$ s^{-1}) initiation rates.

Fig. S7 explores the first case, where TF never blocks NS diffusion but the torsional stress depends on RNAP density. The results are identical to the $f(n) = 1$ case shown in Fig. S5: a fast elongation rate is maintained for *all* initiation rates for both active *and* repressed promoters. This result suggests that without TF blocking NS diffusion (as in linear DNA or less massive TF), increasing torsional stress with RNAP density $f(n)$ is not sufficient to capture the observed cooperative dynamics of RNAPs for the active promoter nor the antagonistic dynamics upon promoter repression.

In the second case, where there is no NS diffusion but with the original $f(n)$, elongation rates drop to zero for all initiation rates and promoter states. This does not exclude the possibility that we can find another $f(n)$ that results in qualitative agreement with experimental observations, even with no NS diffusion. However, our efforts to find such a set of parameters revealed that under no conditions can we simultaneously capture two different phenomena: (i) high elongation rates independent of initiation rates (and hence of RNAP density) in the active state of the promoter and (ii) drastic slow-down upon promoter repression, with lower v_{OFF} for a larger number of RNAPs on the gene. This result implies that LacI, the TF used in the experimental study [13], controls not only RNAP loading events but also the torsional stress of the elongation complexes depending on the ON and OFF states of the promoter. It remains to be tested whether this new role of TF can be found in other TFs or DNA-binding proteins, such as histones in eukaryotic cells.

S4. BURSTY INITIATION

In our model, RNAP loading is assumed to be at regular intervals, motivated by the absence of convoy formation (bursty transcription) under the experimental conditions of [13]. However, [6] proposes that RNAPs loaded close to each other translocate at the same speed and travel as a convoy during elongation. To test this scenario, we explicitly modeled bursty initiation, with 5 RNAPs loading in quick succession within a single burst and a longer duration between bursts. The results are shown in Fig. S8(b) in comparison to nonbursty loading in Fig. S8(a). We find that

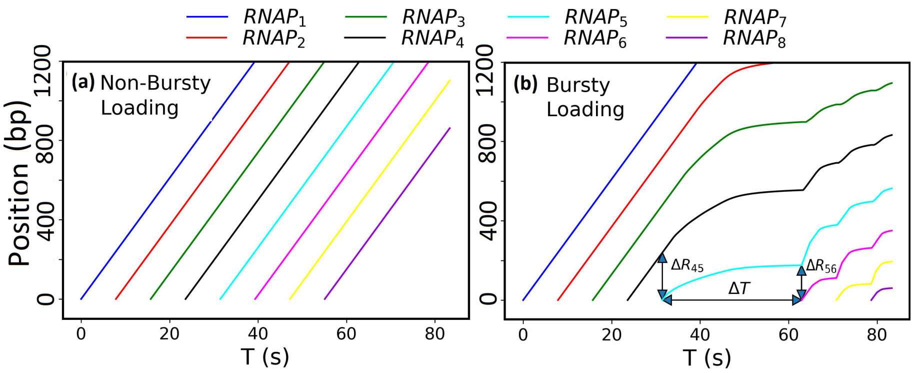

FIG. S8. (color online) Translocation dynamics of RNAPs from nonbursty and bursty initiation. (a) Trajectories of RNAPs from nonbursty initiation at $\alpha = 0.127$ s^{-1}. (b) Trajectories of RNAPs from bursty initiation, where 5 RNAPs are loaded in a burst and $\Delta T > \alpha^{-1}$ is the duration between bursts.

even though the loading of the last RNAP in a burst ($RNAP_5$) and the first RNAP in the next burst ($RNAP_6$) is separated by a longer time duration (ΔT) than the loading of RNAPs within a burst(e.g., $RNAP_4$ and $RNAP_5$), the physical separation between them (ΔR_{56}) is comparable to the separation between RNAPs in a particular burst (ΔR_{45}). Here, the accumulation of NS behind the last RNAP in a burst leads to reducing its speed and thus the physical distance to the RNAP in the next burst. This prediction of our model suggests that convoy formation is hindered even with imposed bursty initiation. Additional mechanisms removing DNA supercoils may help maintain convoys after bursty transcription initiation.
